# Next‐generation phenotyping in cat‐eye syndrome based on computer‐aided facial dysmorphology analysis of normal photographs

**DOI:** 10.1002/mgg3.1785

**Published:** 2021-08-25

**Authors:** Thomas Liehr, Nicole Fleischer, Ahmed Al‐Rikabi

**Affiliations:** ^1^ Jena University Hospital Institute of Human Genetics Friedrich Schiller University Jena Germany; ^2^ FDNA inc Boston Massachusetts USA

## Abstract

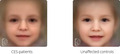

## CONFLICT OF INTEREST

The authors declare that the research was conducted in the absence of any commercial or financial relationships that could be construed as a potential conflict of interest.

In medical genetics even defined clinical syndromes with clear etiology and uniform underlying genetic cause can show large variance in signs and symptoms. This and other factors, like clinician's experience, onsite testing availability or country's reimbursement system influence speed and possibilities to provide diagnoses to patients and their families (Nguyen et al., 2012). Such hurdles are especially true for patients with small supernumerary marker chromosomes (sSMCs). sSMCs are detected by banding cytogenetics in correspondingly specialized laboratories, most often in infertile patients or such with clear clinical abnormalities (Liehr, 2021). Even though several sSMC‐related syndromes are defined, between 1% and 30% of such sSMC carriers show no (or only mild) symptoms, most likely due to mosaicism (Iourov et al., 2019; Liehr, 2021; Liehr & Al‐Rikabi, 2019). This kind of variance is also known for cat‐eye syndrome (CES) patients, presenting an sSMC derived from chromosome 22, first reported in 1965 (OMIM; #115470). Usually CES patients have a karyotype 47,XN,+inv dup(22)(q11.2), leading to a partial tetrasomy of 22pter to 22q11.2. According to literature CES patients have a typical face with coloboma, preauricular pits, and anal atresia. However, the latter three conduction symptoms can be even completely absent (Liehr, 2021; Liehr & Al‐Rikabi, 2019).

CES patients may not have a diagnosis either due to (i) lack of diagnostic capabilities or (ii) as the local diagnostic capabilities are even too advanced. (i) Most of mankind lives in countries with underdeveloped medical systems, where CES patients may in best case get a karyotype, an sSMC is detected, but there are no financial means to further characterize its origin and content (Liehr et al., 2018). (ii) In countries with better medical systems sSMC cases can be solved and CES patients with symptoms will get their diagnoses. In case of mild symptoms due to sSMC mosaicism or in sSMC causing infertility, standard clinical practice tests such cases by molecular karyotyping and/or next‐generation sequencing; here the chance to miss (euchromatic parts of) sSMC is ~80% (Liehr & Hamid Al‐Rikabi, 2018). If in such a case banding cytogenetics as bases test has been skipped, (mosaic) sSMCs are missed and/or results misinterpreted: the centromere‐near tetrasomy 22 may be interpreted as less harmful partial trisomy or intrachromosomal duplication.

A way to overcome these hurdles is applying next‐generation phenotyping (NGP) approaches; therefore just portrait 2D facial photos of a patient are needed, being analyzed by computer vision, and deep learning algorithms that suggest suspected clinical diagnoses (Liehr et al., 2018). Recently we applied NGP with an online tool called Face2Gene (FDNA inc. USA) for the identification of facial phenotypes of two other sSMC‐associated syndromes (Emanuel and Pallister Killian syndrome) and published the results (Liehr et al., 2018). Here, we present the extension of this approach to CES.

**FIGURE 1:**
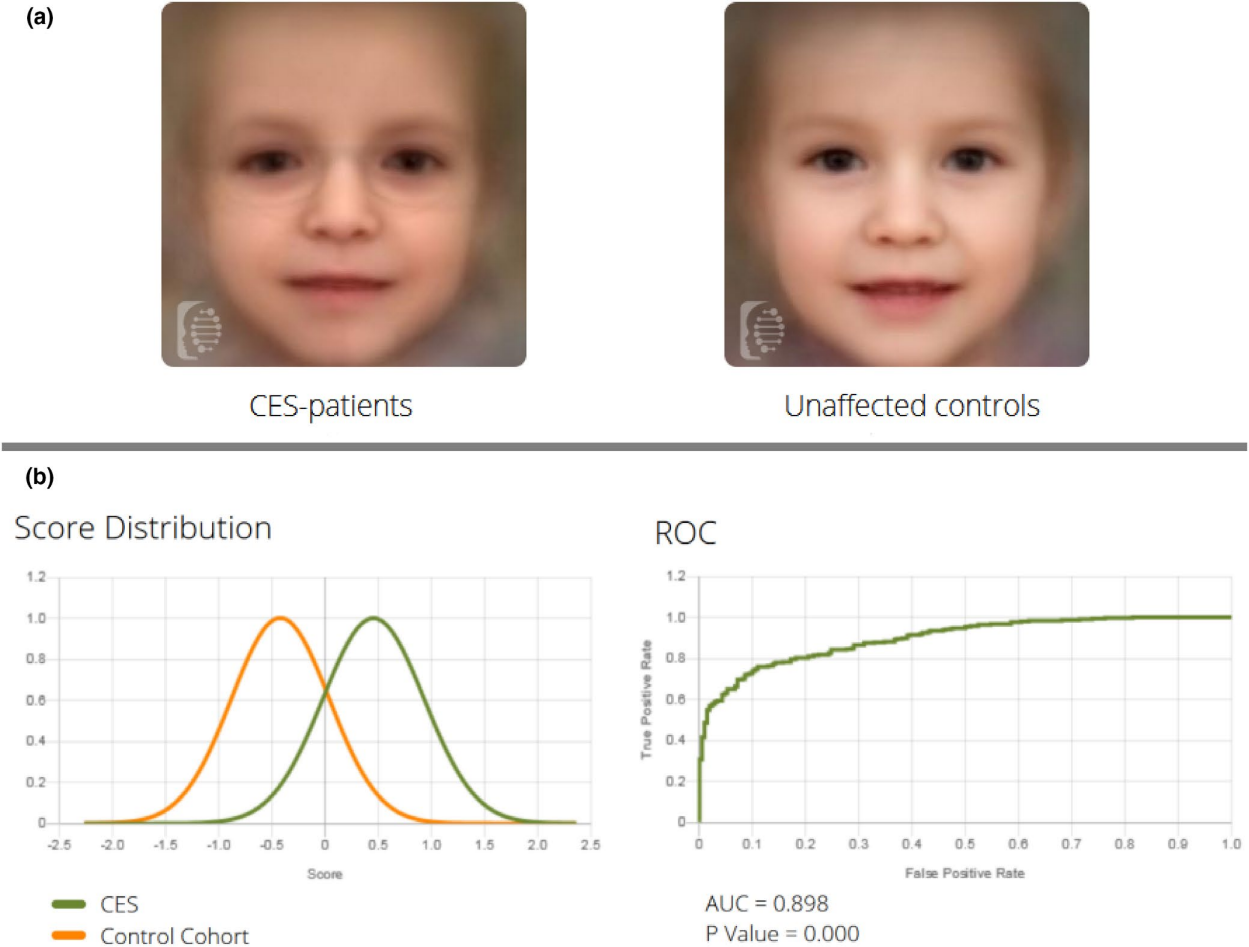
(a) Composite images created for both groups being compared in this study—CES patients and age/sex/ethnicity matched unaffected controls. (b) Score distribution and ROC curve showing the comparison results, displaying and AUC = 0.898 with a *p* value <0.0001

The study (Ethical commission, Friedrich Schiller Universität Jena, Germany—#4738‐03/16 approved) was based on anonymized frontal images (facial gestalt), collected from individuals having either a definite CES diagnosis or considered as clinically normal. Forty‐two images from 27 CES‐patients between 0 and 40 years of age, and 42 images of matched controls were applied (Figure 1). The separation quality (CES vs. controls) was evaluated by creating composite images of both groups (Figure 1a) and measuring the Area Under the Curve (AUC = 0.89) of the receiver operating characteristic (ROC) curve (Figure 1b) as previously described(Liehr et al., 2018). A significant separation between both groups (*p* < 0.0001) leading us to the conclusion that the algorithms can identify the facial phenotype of CES patients, and thus help guide clinicians to the correct type of further laboratory testing needed.

Even though today next‐generation sequencing technologies with DNA‐variant interpretations are clearly “en vogue,” these are not able to solve all problems of medical genetics. sSMCs are evidence proving that cytogenetics still has its place in the concert of cytogenomic approaches. Moreover, NGP technologies, such as the one described here is another, new player in cytogenomics, which needs more attention—more such syndromes need to be included.

## References

[mgg31785-bib-0001] Iourov, I. Y. , Vorsanova, S. G. , Yurov, Y. B. , & Kutsev, S. I. (2019). Ontogenetic and pathogenetic views on somatic chromosomal mosaicism. Genes (Basel), 10, 379. 10.3390/genes10050379 PMC6562967 31109140

[mgg31785-bib-0002] Liehr, T. (2021). Small supernumerary marker chromosomes. http://cs‐tl.de/DB/CA/sSMC/0‐Start.html. Accessed 27, May 2021.

[mgg31785-bib-0003] Liehr, T. , Acquarola, N. , Pyle, K. , St‐Pierre, S. , Rinholm, M. , Bar, O. , Wilhelm, K. , & Schreyer, I. (2018). Next generation phenotyping in Emanuel and Pallister‐Killian syndrome using computer‐aided facial dysmorphology analysis of 2D photos. Clinical Genetics, 93, 378–381. 10.1111/cge.13087 28661575

[mgg31785-bib-0004] Liehr, T. , & Al‐Rikabi, A. (2019). Mosaicism: Reason for normal phenotypes in carriers of small supernumerary marker chromosomes with known adverse outcome. A systematic review. Frontiers in Genetics, 10, 1131. 10.3389/fgene.2019.01131 31781176 PMC6859531

[mgg31785-bib-0005] Liehr, T. , & Hamid Al‐Rikabi, A. B. (2018). Impaired spermatogenesis due to small supernumerary marker chromosomes: The reason for infertility is only reliably ascertainable by cytogenetics. Sexual Development, 12, 281–217. 10.1159/000491870 30089300

[mgg31785-bib-0006] Nguyen, K. T. , Khuat, O. T. , Ma, S. , Pham, D. C. , Khuat, G. T. , & Ruger, J. P. (2012). Impact of health insurance on health care treatment and cost in Vietnam: A health capability approach to financial protection. American Journal of Public Health, 102, 1450–1461. 10.2105/AJPH.2011.300618 22698046 PMC3464830

